# Autosomal-recessive cerebellar ataxia caused by a novel *ADCK3* mutation that elongates the protein: clinical, genetic and biochemical characterisation

**DOI:** 10.1136/jnnp-2013-306483

**Published:** 2013-11-11

**Authors:** Yo-Tsen Liu, Joshua Hersheson, Vincent Plagnol, Katherine Fawcett, Kate E C Duberley, Elisavet Preza, Iain P Hargreaves, Annapurna Chalasani, Matilde Laurá, Nick W Wood, Mary M Reilly, Henry Houlden

**Affiliations:** 1 MRC Centre for Neuromuscular Diseases, UCL Institute of Neurology and National Hospital for Neurology and Neurosurgery, London, UK; 2 Department of Molecular Neuroscience, UCL Institute of Neurology and National Hospital for Neurology and Neurosurgery, London, UK; 3 Section of Epilepsy, Department of Neurology, Neurological Institute, Taipei Veterans General Hospital, Taipei, Taiwan; 4 National Yang-Ming University School of Medicine, Taipei, Taiwan; 5 University College London, Genetics Institute, London, UK; 6 Neurometabolic Unit, National Hospital of Neurology and Neurosurgery, London, UK

**Keywords:** Cerebellar Ataxia, Movement Disorders, Mitochondrial Disorders, Myoclonus, Neurogenetics

## Abstract

**Background:**

The autosomal-recessive cerebellar ataxias (ARCA) are a clinically and genetically heterogeneous group of neurodegenerative disorders. The large number of ARCA genes leads to delay and difficulties obtaining an exact diagnosis in many patients and families. Ubiquinone (CoQ10) deficiency is one of the potentially treatable causes of ARCAs as some patients respond to CoQ10 supplementation. The AarF domain containing kinase 3 gene (*ADCK3*) is one of several genes associated with CoQ10 deficiency. *ADCK3* encodes a mitochondrial protein which functions as an electron-transfer membrane protein complex in the mitochondrial respiratory chain (MRC).

**Methods:**

We report two siblings from a consanguineous Pakistani family who presented with cerebellar ataxia and severe myoclonus from adolescence. Whole exome sequencing and biochemical assessment of fibroblasts were performed in the index patient.

**Results:**

A novel homozygous frameshift mutation in *ADCK3* (p.Ser616Leufs*114), was identified in both siblings. This frameshift mutation results in the loss of the stop codon, extending the coding protein by 81 amino acids. Significant CoQ10 deficiency and reduced MRC enzyme activities in the index patient's fibroblasts suggested that the mutant protein may reduce the efficiency of mitochondrial electron transfer. CoQ10 supplementation was initiated following these genetic and biochemical analyses. She gained substantial improvement in myoclonic movements, ataxic gait and dysarthric speech after treatment.

**Conclusion:**

This study highlights the importance of diagnosing *ADCK3* mutations and the potential benefit of treatment for patients. The identification of this new mutation broadens the phenotypic spectrum associated with *ADCK3* mutations and provides further understanding of their pathogenic mechanism.

## Introduction

Autosomal-recessive cerebellar ataxias (ARCA) are a group of inherited neurodegenerative disorders characterised primarily by cerebellar ataxia, but are frequently associated with other neurological manifestations including spasticity, peripheral neuropathy, seizures and optic atrophy. ARCAs are genetically heterogeneous with more than 20 causative genes currently recognised.^1–3^ Coenzyme Q10 (CoQ10) deficiency (MIM_607426) is one of the potentially treatable causes of ARCA as the symptoms in many patients improve with CoQ10 supplementation.^4^ ^5^ CoQ10 is a lipid-soluble component located in the inner mitochondrial membrane. It plays a pivotal role in the oxidative phosphorylation system (OXPHOS) by shuttling electrons derived from mitochondrial respiratory chain (MRC) complexes I (NADH ubiquinone oxidoreductase) and II (succinate ubiquinone oxidoreductase) to complex III (ubiquinol cytochrome c oxidoreductase), and also participates in other cellular processes as a potent antioxidant, and by influencing pyrimidine metabolism.^6^ ^7^

The AarF domain containing kinase 3 gene (*ADCK3*, MIM_606980) is one of the genes involved in the biosynthetic pathway of CoQ10, thus, their mutations can cause CoQ10 deficiency where most mutations are at loss of function. *ADCK3* is the homologue of the yeast Coq8 gene and encodes a mitochondrial protein which functions in an electron-transferring membrane protein complex in the MRC.^8^ Patients with *ADCK3* mutations usually have disease onset in infancy or early childhood and can present with pure cerebellar ataxia or a complex phenotype with additional features, such as seizures, cognitive impairment, depression, peripheral neuropathy, strabismus or exercise intolerance.^8–11^

In this study, we report two affected siblings in their 20 s, from a consanguineous family of Pakistani origin, who both presented with cerebellar ataxia, myoclonus and dysarthria. Whole exome sequencing (WES) performed in the index patient identified a homozygous frameshift mutation in *ADCK3.* This mutation results in the loss of the stop codon and extension of the protein by 81 amino acids. CoQ10 level and the MRC enzyme activities in the index patient's fibroblasts were also investigated and CoQ10 supplementation was given to her based on the genetic and functional results. Significant improvements in all symptoms mentioned above were observed after treatment.

## Patients and methods

### Patients

With informed consent, we clinically evaluated and obtained blood samples from the index patient and the affected sibling. A skin biopsy was also obtained from the index patient. This study was approved by the joint ethics committee of UCL Institute of Neurology and The National Hospital for Neurology and Neurosurgery, London, UK.

### WES and variant calling

In an attempt to rapidly identify the underlying genetic mutation, WES was carried out on the index patient at the NIH (Bethesda, USA). Nimblegen SeqCap EZ Exome kit (in solution capture) was used for the exome capture. Shotgun sequencing libraries were generated from 3 µg genomic DNA which was extracted from peripheral blood using Flexigene extraction kit and Autopure LS (Qiagen) extraction system. Sequencing was performed on a Genome Analyzer IIx, according to the manufacturer’'s instruction. FASTQ files were aligned to the hg19 reference sequence using Novoalign V.2.07.19, including hard and soft clipping, quality calibration and adapter trimming. Duplicate reads were excluded using the PICARD tool MarkDuplicates. Calling was performed using SAMtools V.0.18 and single sample calling. The resulting calls were annotated with ANNOVAR. Candidate variants were filtered based on function: frameshift, premature stop, non-synonymous or potential splice-altering variants (defined as being within 5 bp of the actual splice site) and frequency (<0.5% in the 1000 Genomes and the National Heart, Lung, and Blood Institute (NHLBI) Exome Sequencing Project database (http://evs.gs.washington.edu/EVS/) for homozygous and compound heterozygous variants, and absent from both of these databases for heterozygous variants). VCF tools were used to annotate gene information for the remaining novel variants. The Integrative Genomics Viewer (IGV) was used to inspect variants.

### Sanger sequencing

Genomic DNAs of the index patient and the affected sibling were extracted and used in the following genetic analysis (DNAs of the parents were unavailable). PCR of exon 15 and the adjacent intronic junctions of the *ADCK3* gene (NM_020247) were performed to validate the identified variant (primers and conditions available on request). PCR products were puriﬁed on Montage PCR 96 Cleanup Plates (Millipore, Bedford, Massachusetts, USA) and used in sequencing reactions with the ABI BigDye Terminator V.3.1 Cycle Sequencing Kit (Applied Biosystems), and subsequently cleaned using BigDye Terminator removal plates (Applied Biosystems). PCR products were resolved on Applied Biosystems 3730XI Sequencer. Computational analyses of mutations were carried out with Sequencher software (Gene Codes Corporation).

### Cell culture

Primary ﬁbroblasts were obtained from a skin biopsy from the index patient. Fibroblast lines from matched healthy controls were kindly provided by Dr Jan-Willem Taanman, UCL Institute of Neurology and the MRC CNMD Biobank, London. The fibroblasts were cultured in Dulbecco's modified Eagle's medium (DMEM) GlutaMAX supplemented with 10% (v/v) heat-inactivated fetal bovine serum and 1% (v/v) penicillin-streptomycin, and they were maintained at 37°C, humidified and 5% CO_2_ in air.

### Quantification of CoQ_10_ levels

CoQ_10_ levels were quantified using a tandem mass spectrometer with a 2975 HPLC (Waters, Manchester, UK) as previously described.^12^ A hexane:ethanol (5:2, v/v) extraction was initially performed and the upper hexane layer was retained for analysis. Samples were resuspended in 50:50 HPLC grade ethanol and methanol. Separation was achieved using a 3 μM Hypersil Gold C4 (150 mm×3 mm, 3 µm) with a Gold C4 guard column (3, 10 mm length, 3 µm) operated at 40°C at a flow rate of 0.4 mL/min. The mobile phases consisted of methanol, 4 mM ammonium acetate 0.1% formic acid and methanol:isopropanol:formic acid (45:55:0.5, v/v/v) containing 5 mM methylamine as an ion pair reagent. Isocratic delivery of the mobile phase ensured elution of the CoQ_10_ methylammonium adduct at approximately 8 min. *D_6_*-CoQ_10_ was used as an internal standard.

### MRC enzyme activities

Activities of MRC complex I, complex II–III, complex IV (cytochrome *c* oxidase) and citrate synthase (CS) were determined in the fibroblast cells according to methods previously described.^13^ Results were expressed as a ratio to CS activity and were normalised against protein. Protein quantification was determined using the Lowry method using bovine serum albumin as a standard.^14^

## Results

### Clinical features

**Figure 1:**
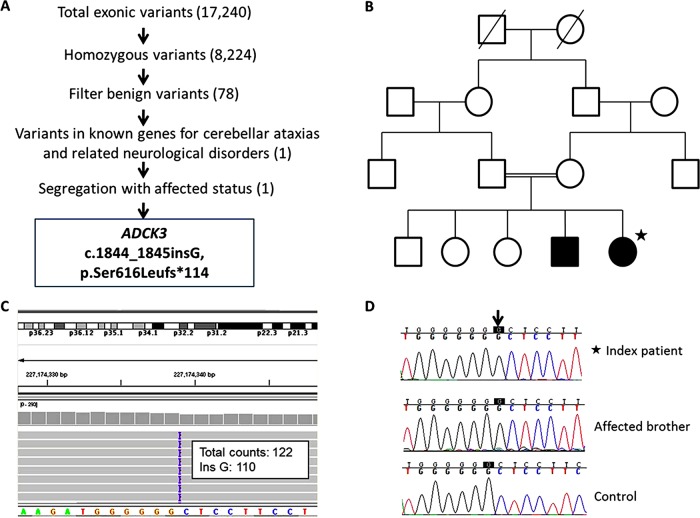
Genetic study and pedigree of the autosomal-recessive cerebellar ataxias (ARCA) family with *ADCK3* S616Lfs mutation. (A) Filtering and prioritisation of variants from whole exome sequencing in the index patient. The criteria of filters were described in the text. (B) Pedigree of this family. The index patient is labelled with a black star. Based on the consanguinity, only homozygous variants were selected. (C) A good sequence depth up to 122× and the homozygosity of the novel *ADCK3* mutation as the insertion G was detected in 110 of the total 122 counts were shown when inspected with the Integrative Genomics Viewer (IGV). (D) Sanger sequencing validated the mutation and proved its segregation with the affected brother.

This 35-year-old lady started to experience myoclonus and jerky tremor in the head and limbs at 10 years old. Slurred speech and unsteady gait developed subsequently in her second decade. There was no response to levodopa. By 30 years of age, she had become wheelchair-dependent because of the ataxic myoclonic gait. She also had difficulty in writing and holding objects due to ataxia and involuntary movements. Her speech fatigued easily, was often tremulous and dysarthric and she also complained of muscle fatigue on exertion. One of the four siblings, currently 32 years old, had a similar presentation with myoclonus, tremor and unsteady gait with the onset at age 14 years. Their parents are first cousins from Pakistan (pedigree shown in figure 1B).

Examination when 35 years old showed cerebellar dysarthria with a tremulous quality to her speech. Eye movements were full with jerky pursuit, marked course tremor of the hands and head, which was both jerky and dystonic in nature. There was upper limb myoclonus, and both upper and lower limb movements were uncoordinated when performing the finger nose task and the heel-knee-shin test. Her gait was ataxic and interrupted by frequent myoclonic jerks and she was unable to tandem walk. Her cognition and visual acuity were normal. She had symmetrical normal reflexes, flexor plantars, normal sensory examination and full muscle power. Her affected brother also had myoclonic movements, ataxic gait and dysarthric speech when examined at age 32 years.

Brain MRI when 35 years old showed mild cerebellar atrophy which was consistent with previous reports of patients with *ADCK3* mutations. Nerve conduction studies and needle electromyography were normal. It is interesting to note the relatively mild phenotype even after 20 years of progressive symptoms. In both affected siblings, acquired causes of ataxia were excluded as was Friedreich's ataxia, ataxia with oculomotor apraxia type 1 (AOA1), ataxia with oculomotor apraxia type 2 (AOA2), ataxia telangiectasia, early onset primary dystonia (DYT1), Vitamin E deficiency, Wilson's disease and spinocerebellar ataxia type 1–3.

### WES and variant validation

The process of variant prioritisation is summarised in figure 1A. A total of 17 240 exonic variants were identified in the index patient. According to the Consensus Coding Sequences hg19 definition of the exome, 92% of exome capture baits had at least 10× depth, and 85% at least 30× depth. After excluding synonymous variants and those with MAF >1%, 78 coding variants remained. These were screened against genes known to be associated with autosomal-recessive cerebellar ataxias and related neurological disorders. A homozygous 1 bp insertion in the *ADCK3* (NM_020247; c.1844_1845insG; p.Ser616Leufs*114) was identified (figure 1C). The variant was validated by Sanger sequencing. Genetic analysis of the affected brother documented the segregation of this variant within the family (figure 1D).

**Figure 2:**
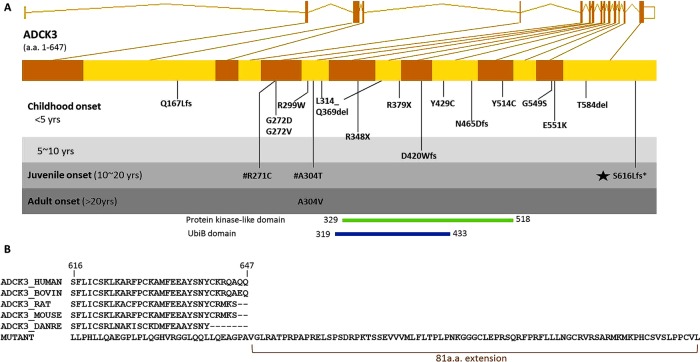
Genomic location of *ADCK3* S616Lfs mutation and the mutant peptide sequence. (A) Genomic structure of the human *ADCK3* gene is shown. All *ADCK3* mutations reported in literature to date are shown in the gene and grouped by the average of patient's age of onset in each family. The novel mutation identified in this study is marked with a black star. #R271C and A304T are two compound heterozygous mutations detected within a previously reported family. D420Wfs, Q167Lfs,Y514C, T584del, L314_Q369del and G549S were reported in Lagier-Tourenne 2008.^8^ E551K, R213W, G272V, G272D and N465Dfs were reported in Mollet 2008.^11^ R348X, R348X and L379X were reported in Gerald 2010.^10^ R271C, A304T, A304V, R299W and Y429C were reported in Horvath 2012.^9^ (B) Multiple species alignment shows this frameshift mutation changes several highly conserved amino acid codons in the terminal segment of *ADCK3* and eliminates the original stop codon, thus extending the peptide by 81 amino acids.

The genomic structure of the human *ADCK3* gene and mutations in this gene are illustrated in figure 2A. These mutations were classified by the patient's age of onset. Our family is the second kindred of *ADCK3* mutation with a juvenile onset of disease (between 10 and 20 years), with a single previous study reporting a family with compound heterozygous missense mutations in *ADCK3*: R271C and A304T.^9^

The novel frameshift we have identified is localised to the C-terminal of *ADCK3*, and results in an alteration of several highly conserved codons in the last coding exon. Furthermore, the frameshift is predicted to eliminate the original stop codon and allow translation to continue on into the three prime untranslated regions (3′UTR), extending the peptide by 81 amino acids (figure 2B).

### CoQ10 level and MRC enzyme activity

The serum CoQ10 level of the patient was found to be within the normal range, however, CoQ10 level in the patient's fibroblast was low—35% of the average CoQ10 level of controls. The assay of MRC enzymes in fibroblasts also revealed that the activities of complex I and complexes II–III were significantly reduced as compared with controls. A decrease in activities of complexes II–III is compatible with CoQ10 deficiency, as the activity of this linked enzyme system is dependent upon endogenous CoQ10.^15^

Details of these analyses are shown in table 1.

**Table 1:** CoQ10 levels and the mitochondrial respiratory chain enzyme activies in the patient with *ADCK3* S616Lfs mutation

	Patient	Control	P/C ratio	p Value
CoQ 10 levels (pmol/mg)				
In serum	43.00	37–133*	/	
In fibroblasts	**31.29**	89.3† (n=50)	**35.0%**	**<0.05**
MRC enzyme activity				
CI/CS	**0.15±0.167**	0.82±0.18 (n=3)	**18.3%**	**<0.05**
CII+CIII/CS	**0.046±0.03**	0.122±0.02 (n=3)	**37.7%**	**<0.05**
CIV/S	0.017±0.0052	0.015±0.004 (n=3)	113.3%	ns

Abnormal values are shown in bold. When experiments were carried out more than once, values are given as means±SE of the mean.

*From the diagnostic laboratory at National Hospital of Neurology and Neurosurgery, Queen Square, London, UK.

†Reference range 57.0–121.6 pmol/mg, average 89.3 pmol/mg; Age: 20.75±1.4 years (range, 0.03–55 years); ratio of males to females, 2:3.^12^

CI, complex I (NADH ubiquinone oxidoreductase); CII, complex II (succinate ubiquinone oxidoreductase); CIII, complex III (ubiquinol cytochrome c oxidoreductase); CIV, complex IV (cytochrome c oxidase: EC 1.9.3.1); CS, citrate synthase; MRC enzyme activity, the mitochondrial respiratory chain enzyme activities assessed in patient's fibroblasts; P/C ratio, the ratio of patient's measurement to control value.

### Treatment and outcome

Following the identification of primary CoQ10 deficiency in the index patient, CoQ10 replacement was initiated with a starting dose of 200 mg twice per day. After 3 months of therapy, her myoclonic symptoms had dramatically improved whereby she was able to discontinue clonazepam which she had been taking for the previous 2 years to manage her frequent myoclonic jerking. The quality of her speech also improved and was less tremulous although there remained some residual dysarthria. Self-reported symptoms of fatigue had also improved.

These improvements were sustained when she was reviewed 6 months after initiation of CoQ10 replacement. There was subjective and objective improvement in her ataxia with a reduction in SARA (scale for the assessment and rating of ataxia, see online supplementary data) scores dropped from 17 to 13 (table 2). Her affected sibling has also shown improvement in speech and fatgue on taking CoQ10 100 mg twice daily for 3 months. The proband and affected brother will be assessed further at 9 and 6 months, respectively, to see if improvement is sustained.

**Table 2:** SARA scores of the patient with *ADCK3* S616Lfs mutation before and after 6 months of CoQ10 supplement

SARA score	Before treatment	After 6 months of treatment
(1) Gait	4	3
(2) Stance	4	3
(3) Sitting	1	1
(4) Speech disturbance	1	1
(5) Finger chase*	2	1
(6) Nose-finger test*	2	1
(7) Fast alternating hand movements*	1.5	1.5
(8) Heel-shin slide*	1.5	1.5
Total	17	13

SARA (scale for the assessment and rating of ataxia) has eight items that yield a total score of 0 (no ataxia) to 40 (most severe ataxia): (1) Gait (score 0–8), (2) Stance (score 0–6), (3) Sitting: (score 0–4), (4) Speech disturbance (score 0–6), (5) finger chase (score 0–4), (6) nose-finger test (score 0–4), (7) fast alternating hand movements (score 0–4), (8) Heel-shin slide (score 0–4).

*Limb kinetic functions (items 5–8) are rated independently for both sides, and the arithmetic mean of both sides is included in the SARA total score.

## Discussion

We demonstrate genetic and biochemical data in a family with a novel frameshift mutation in the *ADCK3* gene and with the phenotype of a complex ataxia-myoclonus syndrome, CoQ10 deficiency and abnormal MRC enzyme activities. One of the unusual features of this family is an onset in the second decade, which is later than most previously reported cases with *ADCK3* mutations. Also, this family was affected with marked myoclonic-dystonic movements but relatively mild cerebellar ataxia, suggesting a wide phenotypic spectrum of *ADCK3* mutations.

To date, autosomal recessive mutations in *ADCK3* have only been identiﬁed in 22 patients from 13 families, and these mutations have been associated with clinically heterogeneous diseases.^9^ Patients usually present with a complex neurological phenotype, with cerebellar ataxia as the predominant manifestation.^8–11^ In this family, cerebellar symptoms were relatively mild compared to the disabling myoclonus and involuntary movements which affected both siblings. This report shows that *ADCK3* mutations should be considered a potential cause of unexplained complex neurological syndromes even when cerebellar ataxia is not the predominant feature.

Unlike previous reports in which the majority of patients had the onset in their childhood or infancy (figure 2),^8–11^ we report the second kindred with a juvenile onset and provide further evidence that *ADCK3* mutations can cause with a variable age at onset.

In our patient, the diagnosis of CoQ10 deficiency was confirmed biochemically by measurement in fibroblasts. Significantly reduced activities of MRC enzymes were also observed in fibroblasts. CoQ10 measurement in muscle is the gold standard for diagnosis of CoQ10 deficiency as plasma concentrations of CoQ10 are not reliable and may be influenced by dietary factors.^12^ However, the measurement in muscle may be normal particularly when the deficiency is mild.^4^ Assessing CoQ10 levels in fibroblasts has been documented to be a reliable method to diagnose CoQ10 deficiency.^12^ ^13^ ^15^ ^16^ Our results confirm that measuring CoQ10 levels in fibroblasts can be an alternative to muscle measurement.

CoQ10 replacement therapy resulted in subjective and objective improvement in patient symptoms and function. The most marked improvements were in reduction of myoclonus and speech improvements although there were also improvements in ataxic symptoms as measured by the SARA scale. CoQ10 deficiency is one of the few treatable causes of ataxia, and symptom improvement following replacement has been observed in a number of patients, although treatment protocols are yet to be standardised.^5^ ^17^ There are few long-term studies, however, and it is not clear for how long these benefits might be sustained, or whether they alter the overall disease course.

The number of patients reported to carry *ADCK3* mutations is still low. This is partly due to the non-specific genotype-phenotype correlations of CoQ10-associated genes and the difficulty in screening all potential genes in suspicious cases. CoQ10 deficiency is associated with clinically heterogeneous diseases, including cerebellar ataxia, encephalomyopathy, severe infantile multisystemic disease, nephropathy, myopathy and multiple-system atrophy, and the number of genes involved in the biosynthetic pathway for CoQ10 is increasing.^4^ ^18^ Our study showed that WES is a powerful tool to detect mutations in rare known genes, particularly in unexplained complex syndromes with high genetic heterogeneity. With the application of WES and other NGS technology, more patients with CoQ10 deficiency are likely to be identified and additional studies may lead to more effective therapies.

The novel mutation identified in this study also provides new insights into the pathogenesis of *ADCK3* mutations. This is the first *ADCK3* mutation which results in the loss of the stop codon and translation into the UTR. CoQ10 deficiency and reduced respiratory enzymes activities in the patient's fibroblast confirmed the pathogenicity of this mutation. It is not yet clear through what mechanism the function of the protein is impaired by the extended C-terminal sequence. The elongated mutant protein may bear less structure stability and thus be incompetent to carry out its function in mitochondria and CoQ10 biosynthesis. However, given the relatively mild disease course, it is likely that some residual function remains.

In summary, our study extends the phenotypic spectrum of *ADCK3* mutations associated with ARCA. Mutations are not only restricted to childhood-onset ataxias, and other neurological features, such as myoclonus, may be prominent. This study also increases the understanding of genetic and functional effects of *ADCK3* mutations. The loss of a stop codon mutation identified in this gene suggests that the terminal segment of *ADCK3* may play an unknown but essential role in its normal function. The great response to treatment in our patient highlights the importance of identifying a potentially treatable cause, CoQ10 deficiency, in unexplained recessive cerebellar ataxia, or ataxia-associated complex syndromes.

## Supplementary Material

Web supplement
